# Better than the Total Variation Regularization

**Published:** 2024-06-21

**Authors:** Gengsheng L. Zeng

**Affiliations:** Department of Computer Science, Utah Valley University Orem, Utah 84058, USA

**Keywords:** Image reconstruction, Total variation prior, Piecewise constant, Limited angle tomography, Gaussian function

## Abstract

The total variation (TV) regularization is popular in iterative image reconstruction when the piecewise-constant nature of the image is encouraged. As a matter of fact, the TV regularization is not strong enough to enforce the piecewise-constant appearance. This paper suggests a different regularization function that is able to discourage some smooth transitions in the image and to encourage the piecewise-constant behavior. This new regularization function involves a Gaussian function. We use the limited-angle tomography problem to illustrate the effectiveness of this new regularization function. The limited-angle tomography situation considered in this paper uses a scanning angular range of 40°. For two-dimensional parallel-beam imaging, the required angular range is supposed to be 180°.

## Introduction

When measurements are insufficient, the image reconstruction problem does not have enough information to obtain a unique reconstruction. For example, the two-dimensional parallel-beam tomography requires a scanning range of 180°. If the data acquisition system does not allow a full scan of 180°, this situation is referred to as the limited-angle tomography, which is almost impossible to obtain a stable reconstruction with the measurements alone. This is a typical compressed sensing problem, which can be solved with additional constraints [1–3].

In X-ray computed tomography (CT), it is reasonable to assume that the images are piecewise-constant. One way to enforce a piecewise-constant image is to enforce the gradient image to be sparse. In theory, the $L_{0}$ ‘norm’ is able to measure the sparseness of an image by counting the non-zero elements if we treat an image as a vector. However, the $L_{0}$ ‘norm’ is not user-friendly in terms of optimization. A convenient alternative remedy is to minimize the total variation (TV) norm of the image.

We put the term norm in single quotes because the *L*_0_ ‘norm’ is not really a norm. In fact, it is not important whether the Bayesian term in the objective function is a norm or not. It is more import that the Bayesian term can effectively characterize the features of the image to be reconstructed. The Bayesian term is a function of the image.

This paper argues that the TV norm is not ideal to enforce the piecewise-constant nature of an image, because the total variation measure cannot distinguish between a smooth monotonic function and a piecewise-constant monotonic function. If a function is monotonically increasing or decreasing on an interval, the total variation value is the absolute value of the difference of the function values at the two end points of the interval.

This paper suggests a new regularization function that is able to distinguish between a smooth monotonic function and a piecewise-constant monotonic function. Our new function contains a Gaussian function, which is an exponential function of a quadratic function.

## Methods

We start with the one-dimensional (1D) case. Let a 1D vector be $x=\left[x_{1},x_{2},\ldots ,x_{n}\right]$. The TV measure for this vector is given as

(1) $$TV(x)=\left|x_{2}-x_{1}\right|+\left|x_{3}-x_{2}\right|+\cdots +\left|x_{n}-x_{n-1}\right|,$$

that is,

(2) $$\text{TV}(x)=\sum_{i=2}^{n}\left|x_{i}-x_{i-1}\right|.$$

The proposed measure is defined below; we call it a Gauss measure because we introduce an exponential factor to each term in (2):

(3) $$\text{Gauss}(x)=\sum_{i=2}^{n}\left|x_{i}-x_{i-1}\right|e^{-\alpha {\left(x_{i}-x_{i-1}\right)}^{2}},$$

where α is a user specified hyperparameter. As α→0, the proposed measure degenerates to the TV measure. Due to the exponential factor in each term of (3), this new measure defined in (3) is not a norm of the vector x because the homogeneity property is violated. If a norm of the vector x is denoted as ‖x‖, the homogeneity property requires that

(2) ‖cx‖=|c|×‖x‖,

for any scalar *c*. The Gaussian factor in (2) destroys this homogeneity property.

**Figure 1:**
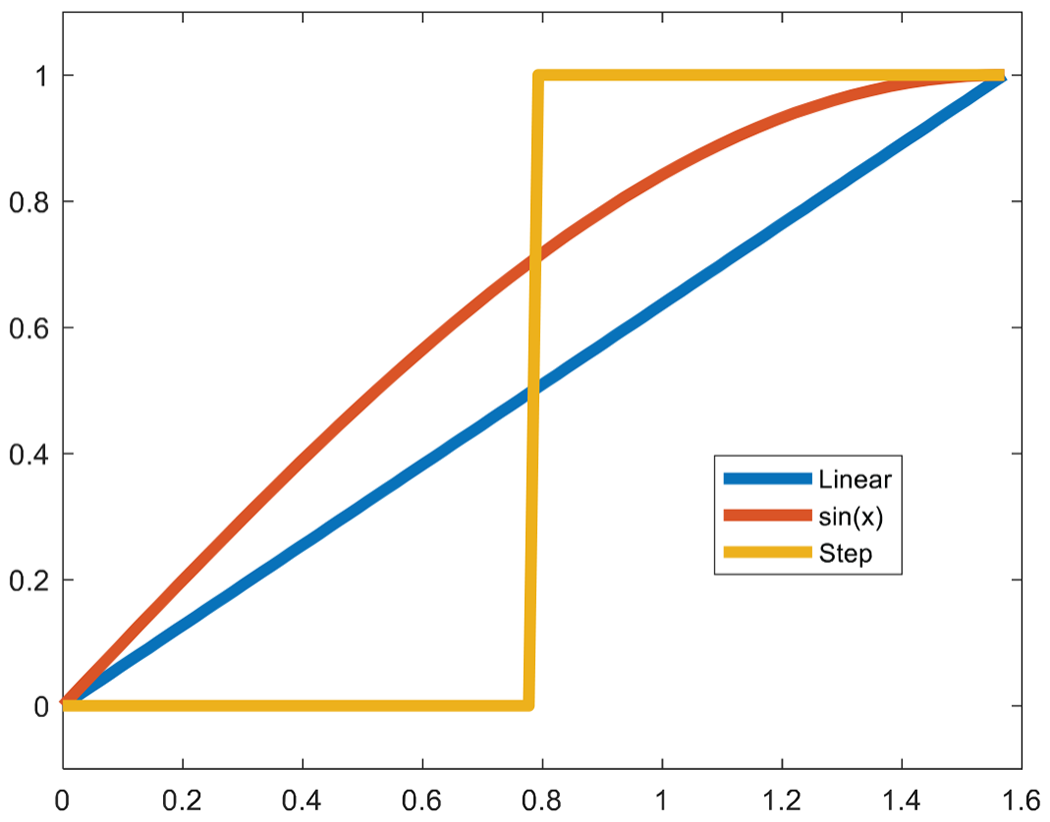
Three functions with the same TV value are defined on [0,π]. The Gauss measures are Linear (blue): 0.9995; Sinewave (red): 0.992; Step (yellow): 0.0067.

Figure 1 shows three functions on the interval [0,π]. The functions are only defined on the 100 points uniformly distributed on [0,π]. Thus, these three functions are, in fact, three vectors. They all have the same TV value of 1.

Let α=5 in (3). The Gauss measure for the linear function (blue) is 0.995, for the sinewave (red) is 0.992, and for the step function (yellow) is 0.0067. Therefore, when (3) is used as the objective function for minimization, the step solution is a preferred solution with the smallest Gaussian measure.

The essence of the Gauss measure defined in (3) is that it encourages a constant region or a large sudden jump. It discourages small gradual changes.

To extend (3) from 1D vectors to images can use the same approaches as to extend the conventional TV norm (2) to images. Usually, two ways are used: isotropic and anisotropic. The anisotropic extension of (3) to the 2D images can be defined as

(4) $$Gauss\left(x\right)=\sum_{i,j=2}^{n}[\left|x_{i,j}-x_{i-1,j}\right|e^{-\alpha {\left(x_{i,j}-x_{i-1,j}\right)}^{2}}+\left|x_{i,j}-x_{i,j-1}\right|e^{-\alpha {\left(x_{i,j}-x_{i,j-1}\right)}^{2}}].$$

Similarly, the isotropic 2 D version can be defined as

(5) $$Gauss\left(x\right)=\sum_{i,j=2}^{n}[\sqrt{{\left(x_{i,j}-x_{i-1,j}\right)}^{2}+{\left(x_{i,j}-x_{i,j-1}\right)}^{2}}\times e^{-\alpha \sqrt{{\left(x_{i,j}-x_{i-1,j}\right)}^{2}+{\left(x_{i,j}-x_{i,j-1}\right)}^{2}}}].$$

As an application of the proposed regularization function (4) or (5), we consider a limited-angle tomography problem. The iterative image reconstruction algorithm is in the form of a maximum-likelihood expectation-maximization (ML-EM) algorithm, similar to that developed in [4]. The gradient of the regularization function is incorporated into the ML-EM algorithm with a small weighting parameter β.

In the computer simulations, the images were 256×256. For a full data set, there were 180 views over 180°. The imaging geometry was parallel beam. The 1D detector had 256 bins. The detector bin size was the same as the image pixel size. The anisotropic 2D version (4) of the image Gaussian measure was adopted in the computer simulations. The proposed MLEM+Gauss algorithm is expressed as

(6) $$x_{i,j}^{\left(n+1\right)}=\frac{x_{i,j}^{\left(n\right)}}{\sum_{k}a_{(i,j)k}+\beta U_{i,j}^{\left(n\right)}}\sum_{k}a_{(i,j)k}\frac{p_{k}}{\sum_{\hat{i},\hat{j}}a_{(\hat{i},\hat{j})k}x_{\hat{i},\hat{j}}^{\left(n\right)}},$$

where $x_{i,j}^{\left(n\right)}$ is the reconstructed image pixel (i,j) at the nth iteration, $p_{k}$ is the kth ray-sum measurement, $a_{(i,j)k}$ is the contribution of pixel $x_{i,j}$ to measurement $p_{k},\beta$ is a control parameter, and $U_{i,j}^{\left(n\right)}$ is the derivative of a penalty function U with respect to the image pixel $x_{i,j}^{\left(n\right)}$ at the nth iteration, that is,

(7) $$U_{i,j}^{\left(n\right)}=\frac{\partial Gauss(x)}{\partial x_{\left(i,j\right)}^{\left(n\right)}}.$$

The associated derivative of (7) is given as

(8) $$U_{i,j}^{\left(n\right)}=\sum_{i,j=2}^{n}\{e^{-\alpha {\left(x_{i,j}-x_{i+1,j}\right)}^{2}}\left[1-2\alpha {\left(x_{i,j}-x_{i+1,j}\right)}^{2}\right]sgn\left(x_{i,j}-x_{i+1,j}\right)+e^{-\alpha {\left(x_{i,j}-x_{i-1,j}\right)}^{2}}\left[1-2\alpha {\left(x_{i,j}-x_{i-1,j}\right)}^{2}\right]sgn\left(x_{i,j}-x_{i-1,j}\right)+e^{-\alpha {\left(x_{i,j}-x_{i,j+1}\right)}^{2}}\left[1-2\alpha {\left(x_{i,j}-x_{i,j+1}\right)}^{2}\right]sgn\left(x_{i,j}-x_{i,j+1}\right)+e^{-\alpha {\left(x_{i,j}-x_{i,j-1}\right)}^{2}}\left[1-2\alpha {\left(x_{i,j}-x_{i,j-1}\right)}^{2}\right]sgn\left(x_{i,j}-x_{i,j-1}\right)\}.$$

## Results

**Figure 2:**
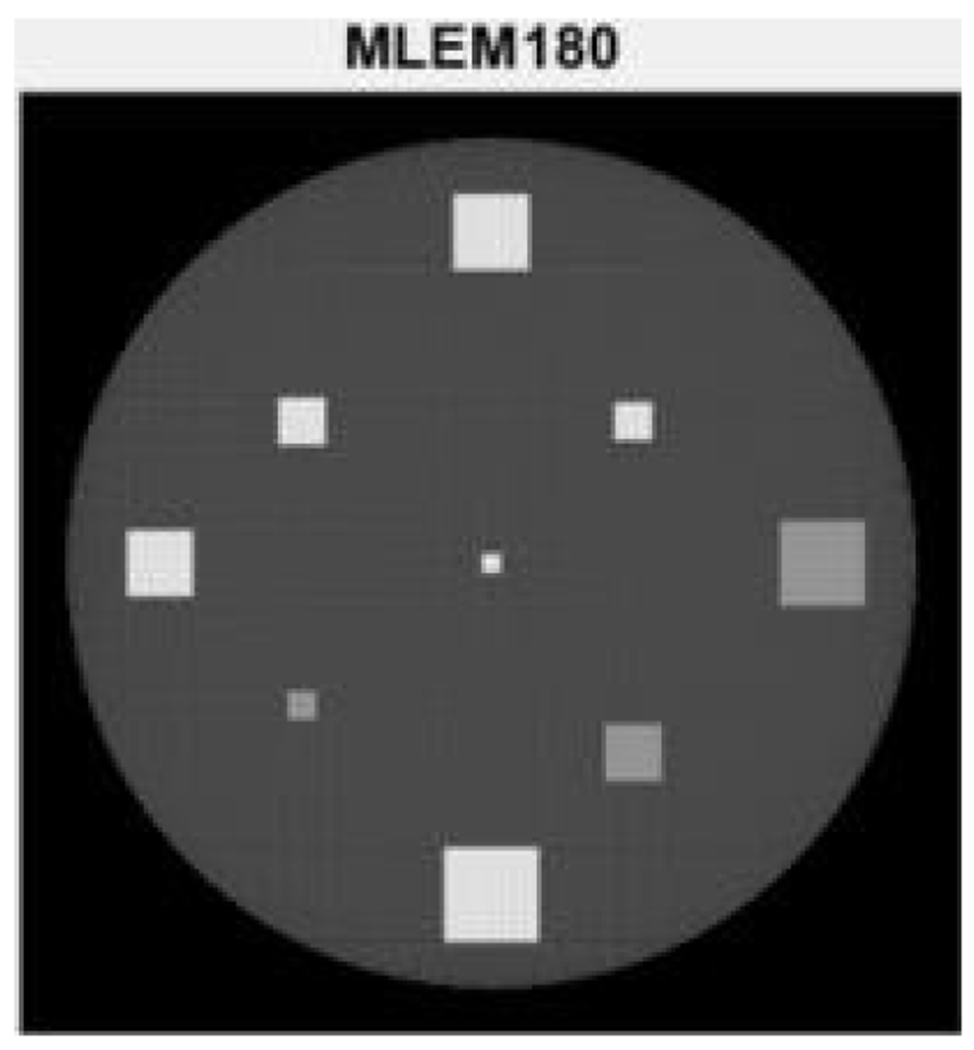
The iterative MLEM reconstruction using the full 180° data set. This image is used as a gold standard for other images to compare with.

**Figure 3:**
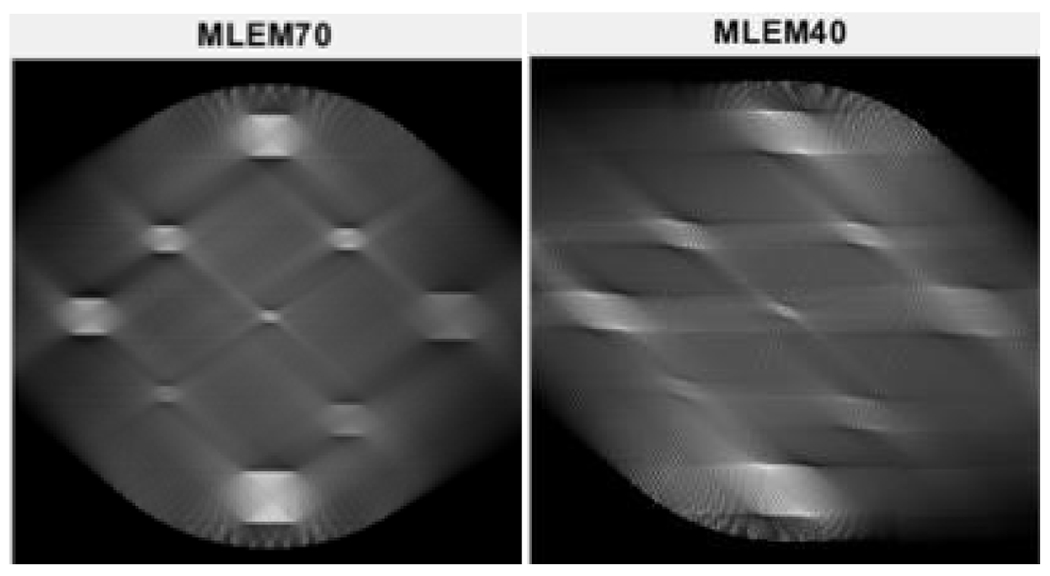
The MLEM algorithm reconstructions when the scanning angular range is (LEFT) 70° and (RIGHT) 40°, respectively.

**Figure 4:**
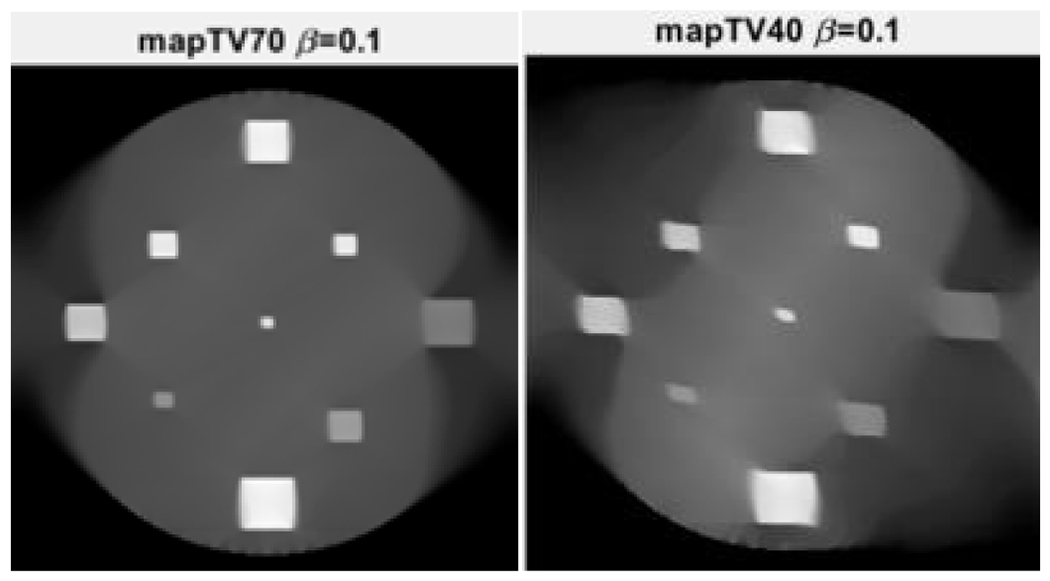
The MLEM+TV algorithm reconstructions when the scanning angular range is (LEFT) 70° and (RIGHT) 40°, respectively.

**Figure 5:**
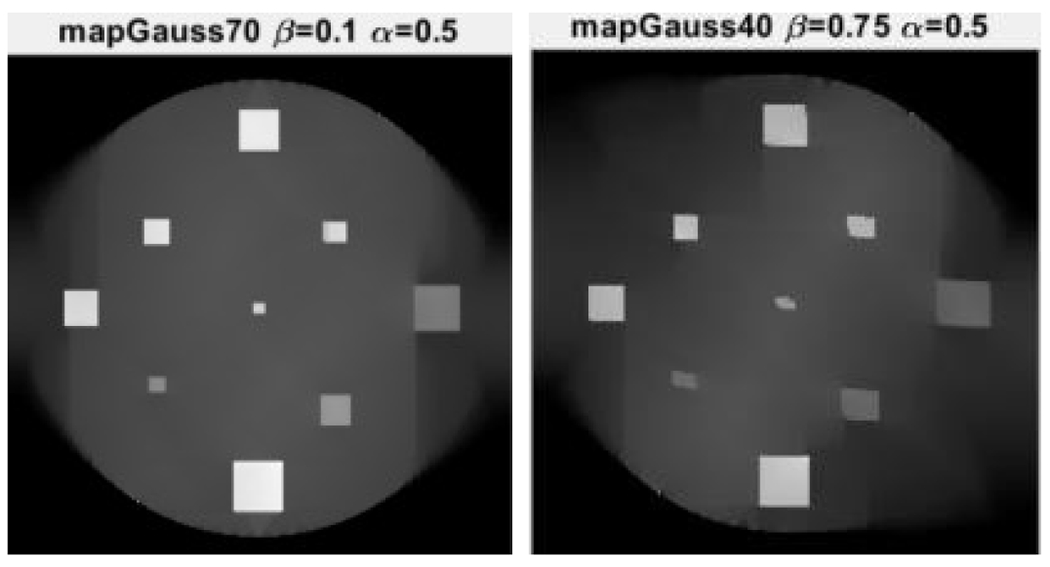
The proposed MLEM+Gauss algorithm reconstructions when the scanning angular range is (LEFT) 70° and (RIGHT) 40°, respectively.

The limited-angle parallel-beam tomography was considered in iterative reconstruction, in which the full angular range is 180°. A 256×256 computer phantom was used, and its MLEM reconstruction using the full range data is shown in Figure 2 as the gold standard for other reconstructions to compare with. The proposed algorithm has two user defined parameters α and β. These parameters were chosen by trial-and-error. The chosen parameters are displayed at the top of Figures 3–5.

Two limited-angle situations were considered: 70° and 40°, respectively, using three algorithms: MLEM, MLEM+TV, and the proposed MLEM+Gauss. The number of iterations was 10,000. Their reconstruction results are shown in Figures 3, 4, and 5, respectively.

It is observed from Figure 3 that the MLEM algorithm is unable to reconstruct any useful images for limited- angle tomography. From Figures 3 and 4, the TV and the proposed Gauss can handle the case of 70° scanning angular range, while the Gauss regularization performs slightly better. When the scanning angular range is further reduced to 40°, the proposed Gauss regularization method clearly outperforms the TV regularization.

## Conclusion

This paper modifies the well-known TV norm by introducing a Gaussian factor. The conventional TV norm has a drawback that it cannot distinguish a smooth function and a piecewise-constant function as illustrated by Figure 1. On the other hand, the newly proposed measure is able to distinguish them. As an application in limited-angle tomography, the proposed method outperforms the TV method when the scanning angular range is as small as 40°. It is expecting that the new regularization method can find many more applications where the measurements are incomplete.
